# Transcriptome analysis of a rice cultivar reveals the differentially expressed genes in response to wild and mutant strains of *Xanthomonas oryzae* pv. *oryzae*

**DOI:** 10.1038/s41598-019-39928-2

**Published:** 2019-03-06

**Authors:** Chunlian Wang, Rezwan Tariq, Zhiyuan Ji, Zheng Wei, Kaili Zheng, Rukmini Mishra, Kaijun Zhao

**Affiliations:** National Key Facility for Crop Gene Resources and Genetic Improvement (NFCRI), Institute of Crop Science, Chinese Academy of Agriculture Sciences (CAAS), Beijing, 100081 China

## Abstract

Bacterial blight (BB), caused by *Xanthomonas oryzae* pv. *oryzae* (*Xoo*), is a devastating disease in most of the rice growing regions worldwide. Among the 42 BB resistance (*R*) genes, *Xa23* is an executor *R* gene, conferring broad-spectrum disease resistance to all naturally occurring biotypes of *Xoo*. In this study, CBB23, a rice line carrying *Xa23* gene, was inoculated with wild PXO99^A^ and its mutant, P99M2, to retrieve the differentially expressed genes (DEGs). RNA-Seq analysis retrieved 1,235 DEGs (*p*-value ≤ 0.05) at 12, 24, 36, and 48 hours of post inoculation (hpi). Gene ontology (GO) analysis classified the DEGs functionally into biological process, cellular component and molecular function. KEGG pathway analysis categorized the DEGs into 11 different pathways, and the ribosome is a prominent pathway followed by biosynthesis of phenylpropanoids. Gene co-expression network analysis identified the clusters of transcription factors (TFs) which may be involved in PXO99^A^ resistance. Additionally, we retrieved 67 differentially expressed TFs and 26 peroxidase responsive genes which may be involved in disease resistance mechanism. DEGs involved in the host-pathogen interaction, e.g., signaling mechanism, cell wall and plant hormones were identified. This data would be a valuable resource for researchers to identify the candidate genes associated with *Xoo* resistance.

## Introduction

Pathogens, i.e., bacteria, nematodes, and viruses, cause widespread losses to agricultural and food commodity on an annual basis. Although plants are commonly in contact with numerous pathogens, the occurrence of disease on an individual plant is relatively intermittent. Plant pathogens have diverse life strategies to weaken the plant defense mechanism^1^.

Bacterial blight (BB), caused by *Xanthomonas oryzae* pv. *oryzae* (*Xoo*), is a devastating disease in most of the rice growing regions. Naturally, *Xoo* enters inside the rice leaf through hydathodes of the leaf margins and multiplies into the intercellular spaces of epithelial tissues, then moves to the xylem vessel for systemic infection^2^. During infection, *Xoo* injects transcription activator-like effector (TALE) protein into the host plant cells via type III secretion system (TTSS) to activate the expression of host genes, contributing to disease development or may activate the resistance (*R*) gene resulting in host defense^3,4^. TALEs are a class of proteins identified in plant pathogenic *Xanthomonas* spp.^3^; basically, a TALE protein is characterized by 34-amino acid central repeat region, an N terminus region for TTSS, and C terminal region containing nuclear localization signal and activation domain^5^. The central repeat region of TALEs identifies the target genes in the host plant cells^5^; each repeat binds to each nucleotide, resulting in a specific binding to the effector binding element (EBE). Furthermore, nucleotide diversity is dogged by the hypervariable position of the 12 and 13 amino acids (denoted as repeat variable diresidues) in each repeat of the TALEs^6^.

To counter the *Xoo* attack, rice plant has developed the defense strategy through a selection of effector binding elements that trap TALEs to activate the expression of *R* genes, triggering host resistance response^7,8^. Until now, 42 *R* genes have been identified^9,10^; among the 42 *R* genes, *Xa23* is an executor *R* gene, which confers broad-spectrum resistance against all naturally occurring *Xoo* biotypes^11^. The expression of *Xa23* results in programmed cell death, or hypersensitive response in plants, exhibiting restriction of pathogen growth and disease resistance phenotype. The expression of *Xa23* is activated by AvrXa23, a TALE protein, present in all *Xoo* field isolates including the highly pathogenic *Xoo* strain PXO99^A^ ^7,11^. In our previous investigations, *Xa23* locus was transferred from wild *Oryza rufipogon* accession (RBB16) to susceptible *indica* rice variety, JG30, resulting in a resistant variety, CBB23^12^; the *avrXa23*-disrupted *Xoo* strain P99M2 has been generated by Tn5-tagged mutagenesis of PXO99^A^ and the mutant P99M2 is virulent in CBB23^13^. Thus, it is hypothesized that the differential response of CBB23 genotype to PXO99^A^ and P99M2 inoculations exists.

In the recent era, the sequencing technologies have become affordable to study the whole transcriptome of an organism in various conditions and at different time periods. RNA-Seq is a revolutionary tool in transcriptomics with high throughput results and low background noise. Additionally, RNA-Seq is considered unbiased technology, used to detect the differentially expressed genes (DEGs) with a broader dynamic range of expression level^14^. The differential response of CBB23 to PXO99^A^ and P99M2 enabled us to study the whole transcriptome of the CBB23 by comparative analysis of the two different *Xoo* strains inoculated leaf samples. In present work, the main objective was to elucidate expression patterns of different genes at different time periods in CBB23 after PXO99^A^ and P99M2 inoculations. After transcriptome analysis, several DEGs were identified; the main emphasis was the functional classification of the DEGs, including peroxidase responsive genes and transcriptional factors, involved in different biological and signaling pathways.

## Results

### Illumina sequencing of rice leaves inoculated with PXO99^A^ and P99M2

**Figure 1:**
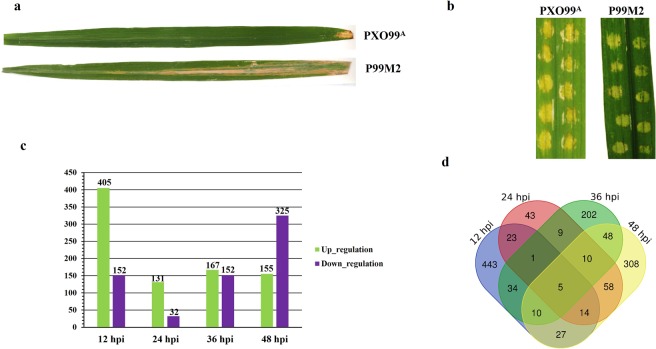
(**a**) Comparative phenotype of the CBB23 leaves after PXOO9^A^ and P99M2 inoculations. Photographs were taken 15 days of post inoculation. (**b**) Inoculations of PXO99^A^ and P99M2 bacterial strains via needleless syringe. Photographs were taken 4 days of inoculation. (**c**) Regulation of DEGs at different time periods in PXO99^A^ inoculated leaves relative to P99M2. Up and down-regulated genes are shown in green and purple color bars, respectively. (**d**) Venn diagram illustrating the overlapping of DEGs at different time points. Overlapping of DEGs at 12, 24, 36, and 48 hpi are shown in blue, red, green and yellow colors, respectively.

Initially, the healthy leaves of CBB23 genotype were infected with PXO99^A^ and P99M2 by scissors dipped method for the confirmation of resistance and susceptibility symptoms (Fig. 1a). Afterward, CBB23 leaves were inoculated by PXO99^A^ and P99M2 via needleless syringe for transcriptome profiling (Fig. 1b). For Illumina sequencing, total RNA from CBB23 leaves of mock (C0) and inoculated samples (PXO99^A^ and P99M2 (12, 24, 36, and 48 hpi)) was extracted to prepare cDNA libraries. The raw data of Illumina sequencing were ranged from 41893788 to 65574380; after filtering the raw sequencing reads containing adapters, Poly-A tail, and low-quality reads, the clean reads were ranged from 40539554 to 62882740 under the 30% Q-phred value (Q-value) (Supplementary Table 1). The sequencing data were sufficient for the transcriptome coverage in rice.

A total of 80% clean reads were mapped to the reference rice genome through Bowtie2 v2.0.0 and TopHat v2.0.12 software allowing a 3-bp mismatch. The 80% clean data mapping to the reference genome enable us that RNA-Seq data is sufficient for subsequent functional annotation and other bioinformatics analysis. The GC contents were ranged from 52.68% to 55.23%. Furthermore, the raw sequencing data of mock, PXO99^A^ and P99M2 inoculated leaves at different time periods have been submitted to NCBI Sequence Read Archive (SRA) which is accessible through the accession number (SRP154928).

### Identification of DEGs in PXO99^A^ vs P99M2 inoculated leaves at different time points

We performed a comparative analysis of DEGs in different sample pairs (PXO99^A^-12 hpi vs P99M2-12 hpi, PXO99^A^-24 hpi vs P99M2-24 hpi, PXO99^A^-36 hpi vs P99M2-36 hpi, PXO99^A^48 hpi vs P99M2-48 hpi) to reveal the expression patterns of genes in CBB23 that play role in resistance to PXO99^A^. The *p*-value ≤ 0.05 and log2 fold change (log2FC) ≥1 or ≤*−*1 were set as a threshold level to retrieve the DEGs in CBB23 at different time points and nominated DEGs were used for further analysis. A total 1,235 DEGs were identified in CBB23 at different time intervals (12, 24, 36, 48 hpi) in PXO99^A^ vs P99M2 (Supplementary Table 2). Among the 1,235 DEGs, 195 genes are hypothetical genes whose actuality are to be revealed by detailed wet lab experiments. The comparative analysis showed that there were more up-regulated genes in PXO99^A^ inoculated leaves than that of P99M2. Briefly, we identified 557 (405 up and 152 down-regulated), 163 (131 up and 32 down-regulated), 319 (167 up and 152 down-regulated) and 480 (155 up and 325 down-regulated) DEGs at 12, 24, 36 and 48 hpi, respectively in PXO99^A^ vs P99M2 (Fig. 1c). Comparatively, 12 hpi sample has the maximum number of DEGs than those of other time periods. Moreover, Venn diagram exposed the overlapping DEGs at different time points (Fig. 1d); only five DEGs were overlapped in all four time periods. Besides, 443, 43, 202 and 308 DEGs were overlapped at 12, 24, 36 and 48 hpi, respectively in CBB23 genotype.

### Identification of differentially expressed TFs

**Figure 2:**
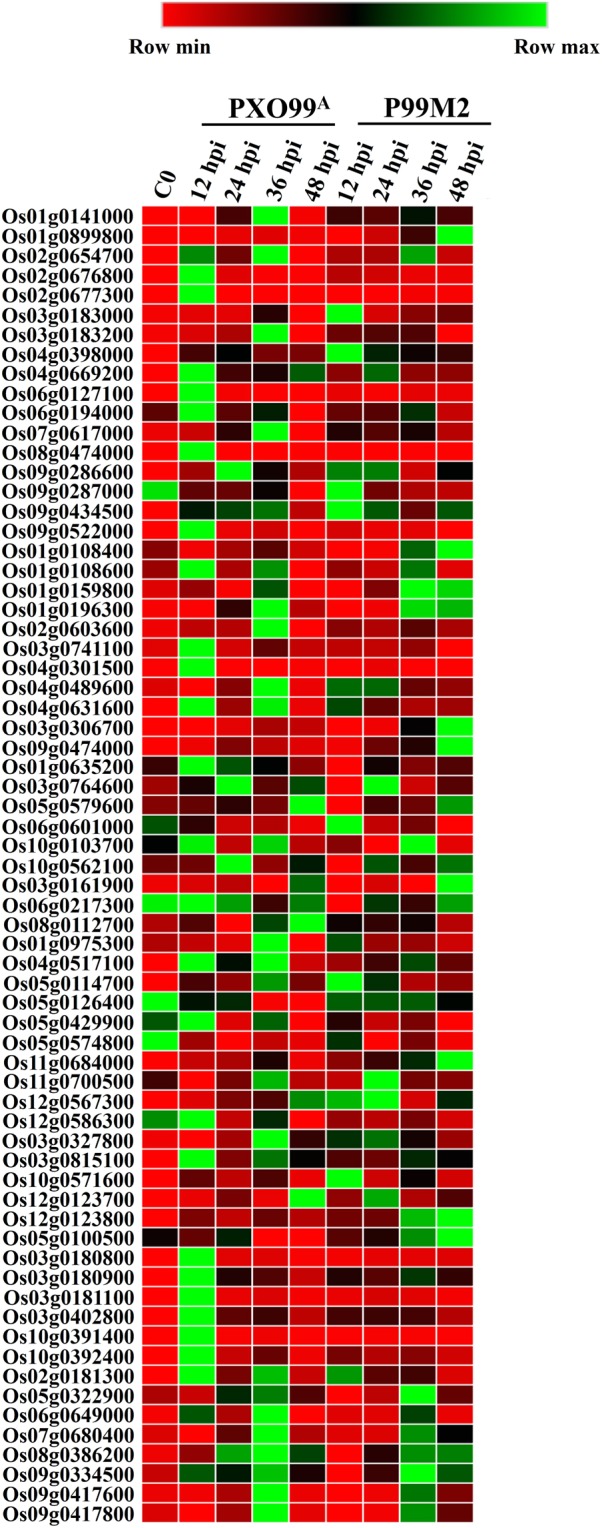
Differentially expressed TFs in CBB23 after inoculation of PXO99^A^ and P99M2 at different time points. Gene IDs and name of TFs were retrieved from RAP-DB and cross-checked to plant TFDB.

Transcription factors (TFs) may activate or repress the genes by binding to the promoter site of the downstream genes in a sequence-specific manner^15^. In our RNA-Seq data, among the 1,235 DEGs, 67 DEGs belonging to 12 different TF families were identified to be differentially regulated, which may have played important role in *Xa23* functioning. (Fig. 2; Supplementary Table 3). Among 47 differentially expressed TFs at 12 hpi, eight AP2-ERF, three bHLH (Os01g0108600, Os03g0741100, and Os04g0301500), five HD, one MADS (Os06g0217300), three MYB (Os04g0517100, Os05g0429900, and Os12g0586300), one NAC (Os03g0815100), and five WRKY TFs were up-regulated in PXO99^A^ vs P99M2. Tify TFs (Os03g0180800, Os03g0180900, Os03g0181100, Os03g0402800, Os10g0391400, and Os10g0392400) were found to be up-regulated at 12 hpi in PXO99^A^ vs P99M2. At 24 hpi, one bHLH (Os01g0108400) and four WRKY TFs (Os05g0322900, Os07g0680400, Os09g0417600, and Os09g0417800) were up-regulated in PXO99^A^ inoculated leaves than that of P99M2. At 36 hpi, 13 differentially expressed TFs (10 up-regulated and 3 down-regulated) were identified; among 13 TFs, four AP2-ERF (Os03g0183200, Os07g0617000, Os09g0286600, and Os09g0287000), two bHLH (Os02g0603600 and Os04g0631600), three MYB (Os01g0975300, Os05g0114700, and Os11g0700500), one WRKY (Os02g0181300) belonged to up-regulated genes. Furthermore, at 48 hpi, only two TFs, one MADS (Os08g0112700) and one NAC (Os12g0123700), were up-regulated out of 20 differentially expressed TFs. Additionally, out of 67 differentially expressed TF, 43 genes were up-regulated, and 24 genes were down-regulated in PXO99^A^ vs. P99M2. Hence, up-regulated TFs in PXO99^A^ inoculated leaves relative to P99M2 may be involved in enhancing the rice immunity.

### Identification of peroxidases related DEGs

**Figure 3:**
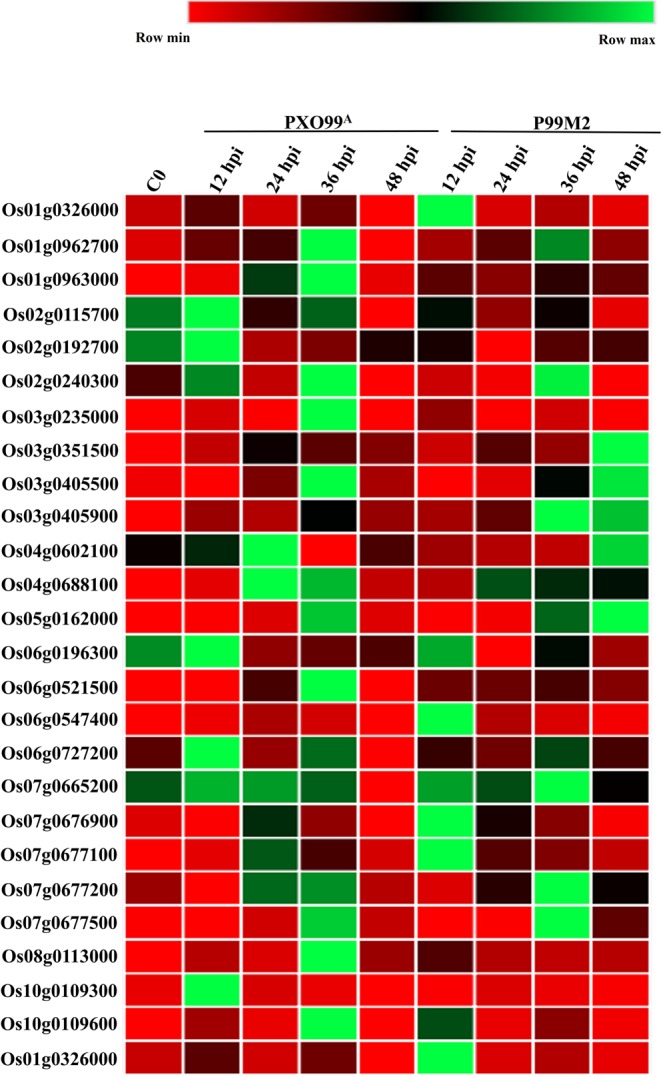
Heat map showing the peroxidase responsive DEGs identified to be differentially regulated at different time points in CBB23 after PXO99^A^ and P99M2 inoculation. The gene IDs of selected Peroxidase responsive genes were retrieved from RAP-DB.

Peroxidases are identified to be involved in cell wall metabolism, wound healing, removal of H_2_O_2_ and toxic reductants^1,16^. In our experiment, 26 peroxidase responsive genes were identified expressed differentially at different time periods (Fig. 3; Supplementary Table 4). Among the 26 DEGs, 17 were down-regulated and 9 up-regulated. At 12 hpi, 4 DEGs (Os02g0192700, Os02g0240300, Os06g0727200, and Os10g0109300) were up-regulated, and 11 DEGs were down-regulated in PXO99^A^ inoculated CBB23 leaves compared to P99M2. At 24 hpi, six DEGs (Os01g0963000, Os02g0240300, Os04g0602100, Os06g0196300, Os07g0677100, and Os07g0677500), were up-regulated in PXO99^A^ vs P99M2. However, at 36 hpi, DEGs, including Os01g0963000, Os03g0235000, Os08g0113000, and Os10g0109600), were up-regulated. Contrary, DEGs at 48 hpi were down-regulated in PXO99^A^ inoculated leaves than that of P99M2. The down-regulated genes may be negatively regulated the CBB23 resistance to PXO99^A^. Given the expression pattern of peroxidase responsive DEGs, it is depicted that peroxidase responsive genes play their role in early infection from 12 to 36 hpi.

### GO analysis of DEGs

GO analysis was done to functionally categorize the DEGs into three groups, i.e., Biological process, Cellular component and Molecular function. The GO analysis was done using AgriGO online tool. GO classification of all the DEGs in CBB23 challenged by PXO99^A^ and P99M2 at different time intervals are mentioned in Supplementary Table 5. To investigate the effect of PXO99^A^ and P99M2 in CBB23 genes expression, the DEGs were analyzed in terms of GO at specific time point using the false discovery rate (FDR) ≤0.05 (Supplementary Table 6). In PXO99^A^ vs P99M2 at 12 hpi, DEGs were divided into 38 GO terms, including five different biological processes, 17 cellular component, and 16 molecular function related terms (FDR ≤0.05). At 24 hpi in PXO99^A^ vs P99M2, 26 significant enriched GO terms were identified, involved in five biological processes, 14 cellular components and seven molecular functions related GO terms. Furthermore, DEGs were significantly enriched in 37 GO terms at 36 hpi; in addition, identified 37 GO terms belong to biological process (6), cellular component (23) and molecular function (8). At 48 hpi in PXO99^A^ vs P99M2, 50 GO terms (FDR ≤ 0.05), comprising 16 biological process, 22 cellular component and 12 molecular function.

**Figure 4:**
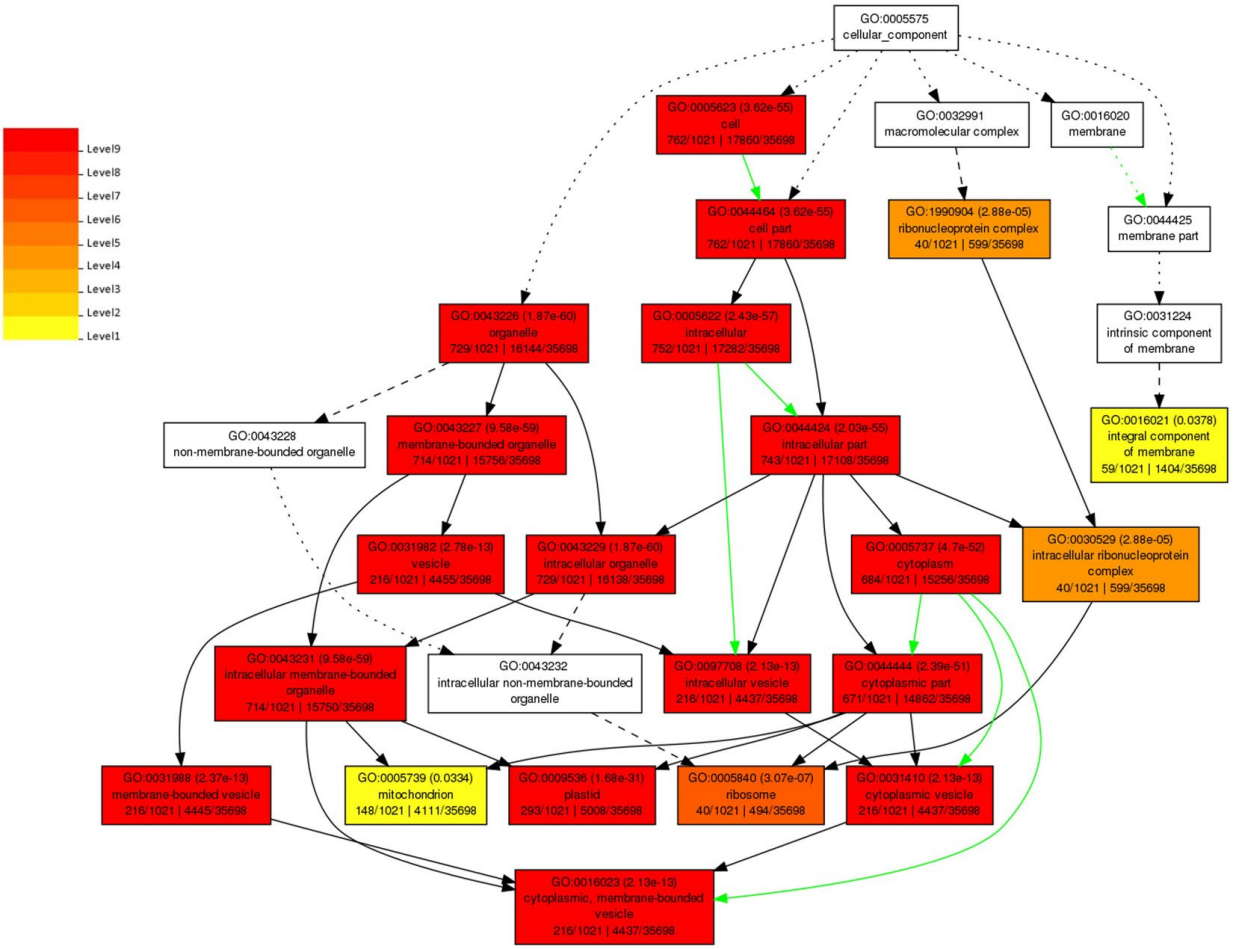
Cellular component related GO terms in CBB23 genotype representing the significant clustering of DEGs in different coloring patterns. A key is shown beside GO terms, which exhibits the significance level of the GO terms.

Similarly, GO analysis was conducted to explore the cellular component related GO terms, significantly enriched in all-time points (12, 24, 36, 48 hpi) (Fig. 4). The significant cellular component GO terms are described as follows: “Cell (GO:0005623)”, “Cell part (GO:0044464)”, “ribonucleoprotein complex (GO:1990904)”, “Organelle (GO:0043226)”, “intracellular (GO:0005622)”, “membrane-bounded organelle (GO:0043227)”, “intracellular part (GO:0044424)”, “integral component of membrane (GO:0016021)”, “vesicle (GO:0031982)”, “intracellular organelle (GO:0043229)”, “cytoplasm (GO:0005737)”, intracellular ribonucleoprotein complex (GO:0030529)”, “Intracellular membrane-bounded organelle (GO:0043231)”, “intracellular vesicle (GO:0097708)”, “cytoplasmic part (GO:0044444)”, “membrane-bounded vesicle (GO:0031988)”, “mitochondrion (GO:0005739)”, “plastid (GO:0009536)”, “ribosome (GO:0005840)”, “cytoplasmic vesicle (GO:0031410)”, and “cytoplasmic, membrane-bounded vesicle (GO:0016023)”.

### Pathway analysis

**Figure 5:**
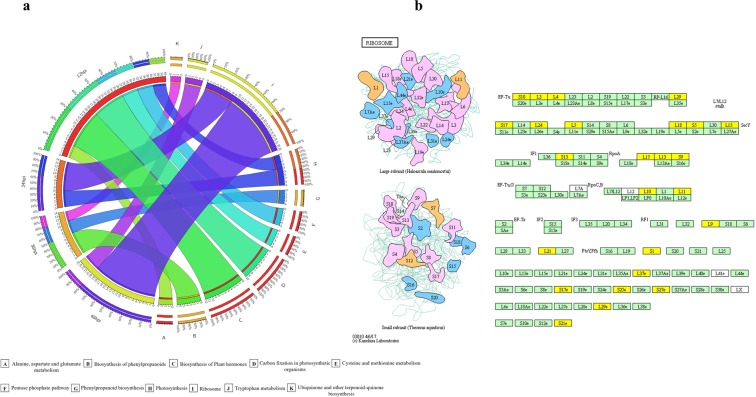
KEGG pathway analysis of DEGs at different time points. (**a**) Circos exhibiting the significant (P-value ≤ 0.05) KEGG pathways at different time intervals. All the significant KEGG pathways are mentioned in different color ribbons. (**b**) KEGG pathway analysis related to the ribosome in the comparison of the PXO99^A^ and P99M2 inoculated rice leaves. Green shade boxes indicate the genes expressing in rice; while yellow boxes are representing the up-regulated DEGs in PXO99^A^ relative to P99M2 inoculation.

The DEGs were mapped on the KEGG pathway to retrieve the significant pathway. The DEGs were involved in different pathways shown in Supplementary Table 7. The KEGG pathways of the DEGs were selected based on *p*-value ≤ 0.05. “Carbon fixation in photosynthetic organisms”, “biosynthesis of the plant hormones”, and “ribosomes” were the significant KEGG pathway in PXO99^A^ inoculated leaves than that of P99M2 at different time points (12, 24, 36, 48 hpi) (Fig. 5a). Briefly, “carbon fixation in photosynthetic organisms”, “pentose phosphate pathway”, “biosynthesis of plant hormones”, and “cysteine and methionine metabolism” were enriched pathway at 12 hpi. Unlike 12 hpi, “ribosome” and “photosynthesis” were found to be significantly enriched pathway at 24 hpi. Moreover, “biosynthesis of phenylpropanoids” was the enriched pathway at 36 hpi; while “ribosome” with 20 DEGs was accounted as the most prominent pathway at 48 hpi. Ribosome related KEGG pathway is shown representing the role of DEGs at different points (Fig. 5b). The large ribosomal subunit responsive genes, i.e., L3, L4, L5, L9, L10, L11, L13, L15, L17, L18, L21, L24, L29, L29e, and L37e were up-regulated. Apart from larger subunit responsive genes, the DEGs involved in the smaller subunit, i.e., S1, S10, S13, S17, S17e, S21e, S25e, and S27e seemed to be up-regulated. The DEGs involved in ribosome related KEGG pathway may be important for resistance mechanism against PXO99^A^ comparatively to the P99M2 strain.

**Figure 6:**
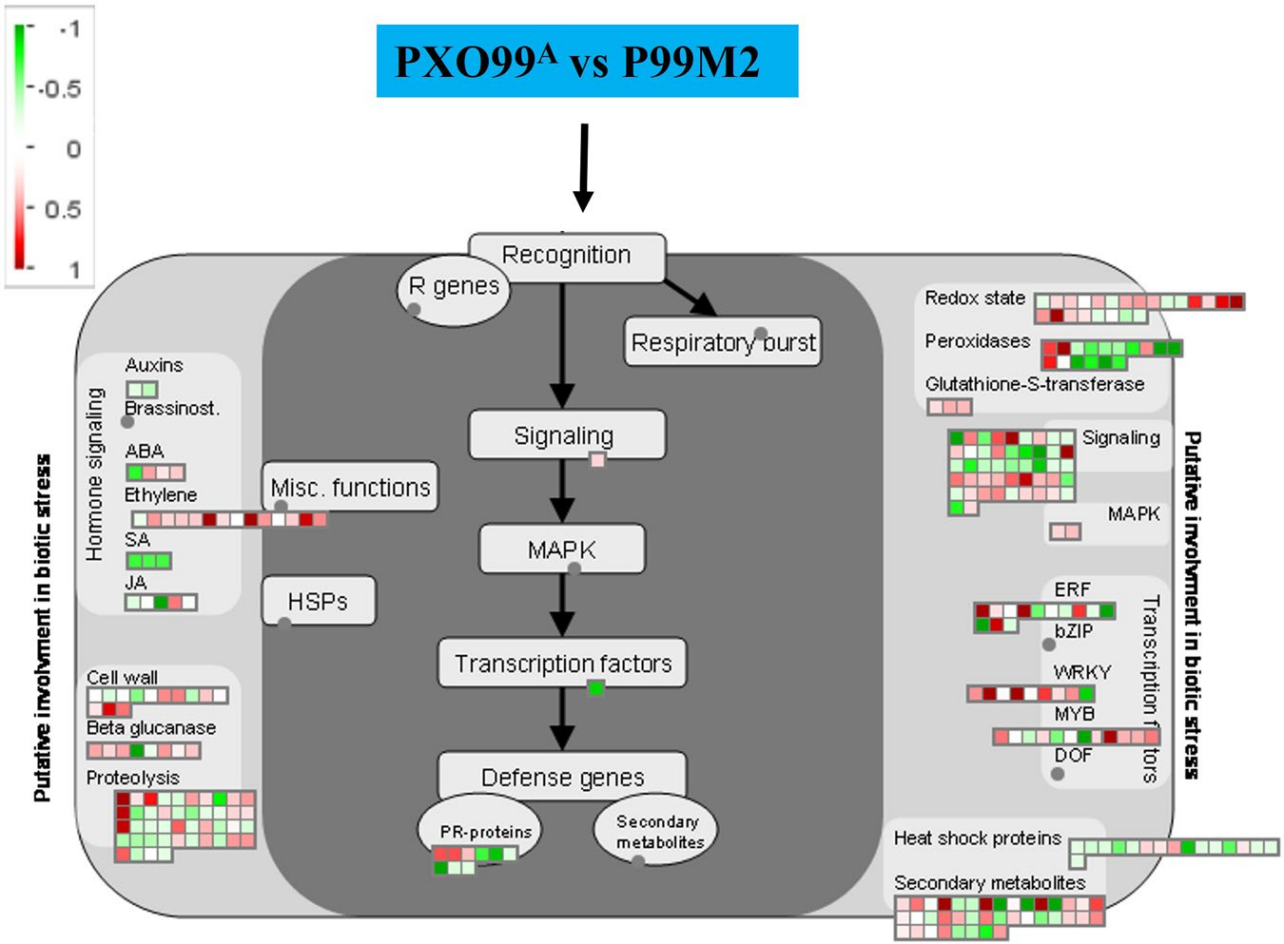
MapMan analysis illustrating the DEGs involved in host-pathogen interaction. DEGs with Log2FC ≥ 1 or ≤*−*1 were imported into MapMan tool. Up and down-regulated genes are represented in red and green squares, respectively.

Furthermore, MapMan package was used to investigate the DEGs, present in plant-pathogen interaction. The DEGs were used as input to curate the specific biological pathway by getting the information from available rice genome database. The DEGs, having known functions in defense, e.g., TFs, peroxidases, kinases, and secondary metabolites were identified (Fig. 6). Most of the DEGs related to the different biological pathways, including signaling, MAPK, ERF, WRKY, MYB and secondary metabolites are up-regulated; this indicates that these up-regulated genes may be played a key role in resistance against PXO99^A^. Four out of 16 different heat shock proteins were identified as up-regulated and expression level was influenced by PXO99^A^ and P99M2. Moreover, 17 secondary metabolites and three pathogenesis-related (PR) genes were identified as up-regulated which may be important in resistance. DEGs related to the auxin, abscisic acid (ABA), ethylene (ET), salicylic acid (SA) and jasmonic acid (JA) were identified; mainly, two DEGs encoding auxin, three SA, and four JA were identified to be down-regulated in CBB23 after PXO99^A^ infection relative to P99M2. 14 DEGs encoding cell wall were identified; seven of 13 call wall-related genes were up-regulated. Moreover, six β glucanases and 21 proteolysis responsive genes were up-regulated in PXO99^A^ compared to P99M2 inoculated leaves. The detail description of the DEGs corresponding to the MapMan analysis are given in Supplementary Table 8. The visual annotations of the DEGs is a valuable resource for the investigation of the pathways involving *Xa23* gene.

### Co-expression network analysis

**Figure 7:**
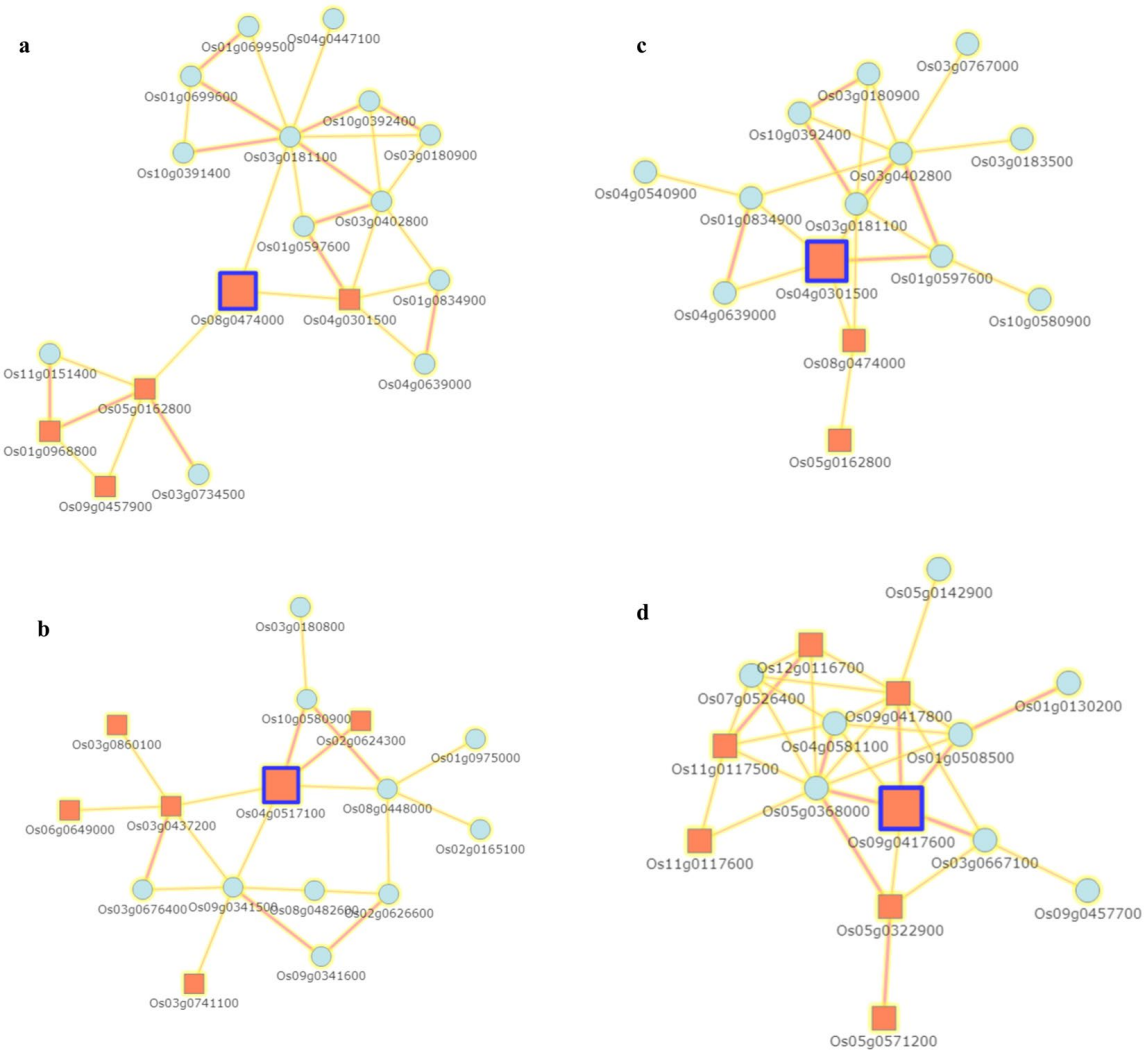
Co-regulated gene expression network analysis of highly expressed TFs. The differentially expressed TFs, i.e., AP2/ERF, bHLH, MYB, and WRKY were retrieved from RNA-Seq data for network analysis; orange color boxes represent the TFs and light blue color circles designate the different rice genes, respectively retrieved from RAP-DB.

The co-expression analysis is important to elucidate the role of DEGs in *Xa23-*mediated resistance against BB. The co-regulation networks of highly expressed TFs are shown in Fig. 7. The interaction scores range from 0.5 to 0.9; high interaction score represents the high confidence interaction (Supplementary Table 9). The network revealed the co-expression of the Os08g0474000 (AP2/ERF104) with Os04g0301500 (OsbHLH6), Os03g0181100, and Os05g0162800 (Fig. 7a). MYB TFs have been found to play role in the biochemical process, plant defense, plant development and secondary metabolism. OsMYB4 (Os04g0517100), interacted with Os10g0580900, Os02g0624300 (OsMYB30), Os09g0341500, Os08g0448000 (Os4CL5) and Os03g0437200 (ZFP36) (Fig. 7b); similarly, bHLH (basic helix-loop-helix) TFs constitute the second largest family in angiosperms; bHLH has been identified to be important for plants against abiotic and biotic stresses. In our network analysis, Os04g0301500 (OsbHLH6), encoding the bHLH TF was found to interact with Os01g0597600 (OsATL15), Os03g0402800 (OsTIFY10), Os01g0834900, Os08g0474000 (OsERF104), and Os04g0639000 (Fig. 7c). Apart from AP2, MYB and bHLH TFs, OsWRKY76 (Os09g0417600) TF, having zinc finger domains exhibited interactions with Os05g0368000 (RH1), Os09g0417800 (OsWRKY62), Os01g0508500 (RH2), Os03g0667100 (OsNPR3), Os05g0322900 (OsWRKY45), and Os04g0581100 (OsS3H) (Fig. 7d). The predicted interaction of TFs with different proteins could be useful to explain the involvement of DEGs in the resistance mechanism against BB.

### Data validation

**Figure 8:**
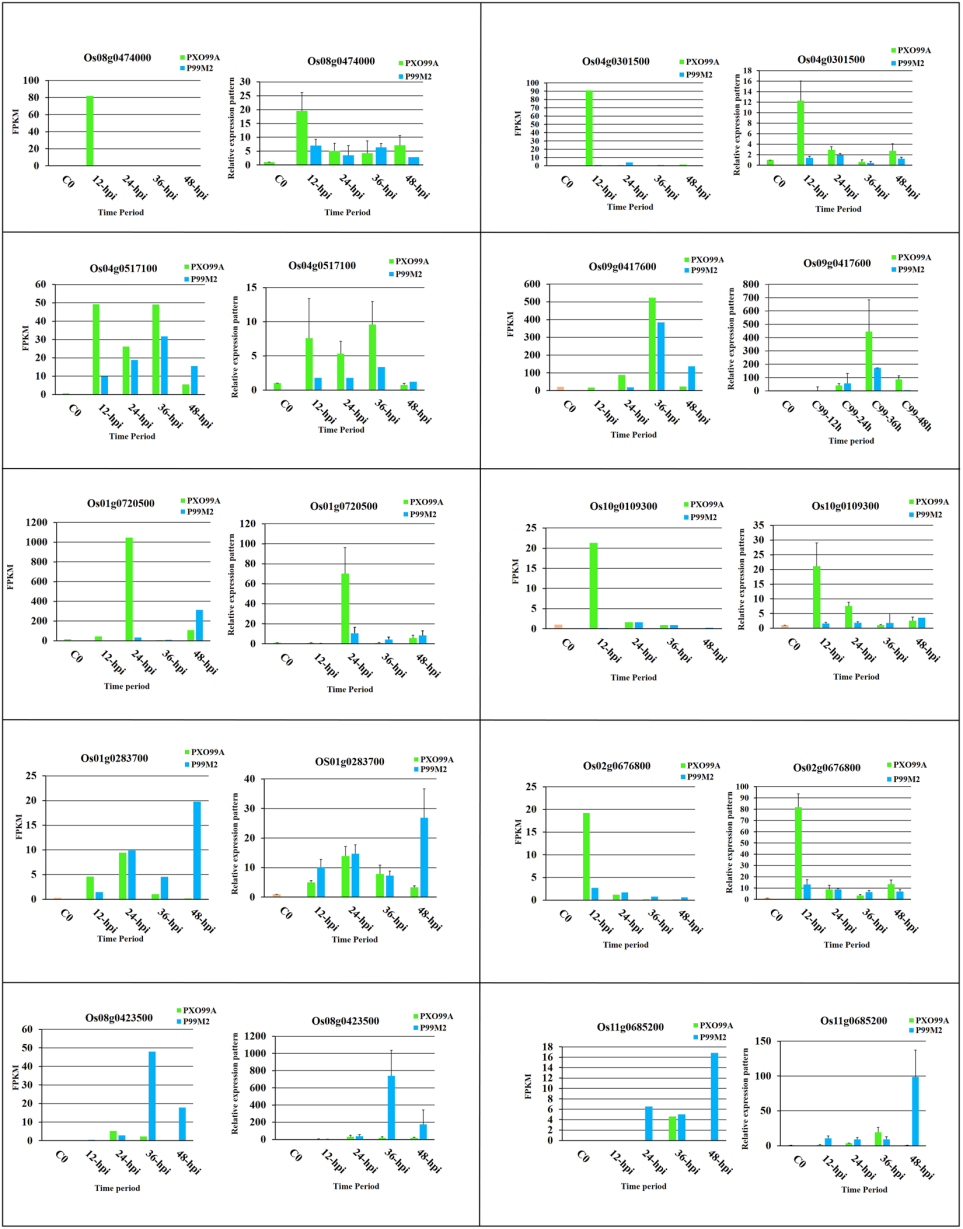
Validation of relative expression patterns of DEGs by qRT-PCR. DEGs were randomly selected from RNA-Seq data for qRT-PCR based validation, and ubiquitin was used as internal control in reaction. Data are represented as mean ± SD of three biological replicates, and RNA-Seq based expression pattern of different genes is represented by FPKM values.

RNA-Seq data were validated by quantitative real-time polymerase chain reaction (qRT-PCR) of the randomly selected DEGs (Fig. 8); DEGs sequences were retrieved from phytozome v12.1. Os08g0474000 and Os04g0301500, encoding *OsERF104* and *OsbHLH6* TFs were highly expressed at 12 hpi in PXO99^A^ inoculated leaves of CBB23 than that of P99M2. Moreover, Os04g0517100, encoding *OsMYB4* was up-regulated from 12 to 36 hpi in PXO99^A^ vs P99M2; *OsWRKY76* (Os09g0417600) was exhibited to be up-regulated at 36 hpi in PXO99^A^ vs P99M2. Os10g0109300 and Os01g0720500 identified as peroxidase and type I chlorophyll a/b binding protein were highly expressed at 12 and 24 hpi in PXO99^A^ vs P99M2, respectively. Os01g0283700, representing the cinnamoyl-CoA reductase was down-regulated in PXO99^A^ vs P99M2 at 48 hpi. Dehydration responsive gene (Os02g0676800), was up-regulated at 12 hpi in PXO99^A^ compared to P99M2. Carbonic anhydrase (Os08g0423500) and a hypothetical gene (Os11g0685200) were up-regulated in 36 and 48 hpi of P99M2. In short, qRT-PCR endorsed the expression pattern of DEGs exhibited by RNA-Seq.

## Discussion

In this study, two *Xoo* isolates, PXO99^A^ and P99M2, were used to study the DEGs involved in resistance pathways underlying *Xa23*. We identified 1,235 DEGs in CBB23 genotype in the comparative analysis after PXO99^A^ and P99M2 infection at different time points. A clear morphological distinction of PXO99^A^ and P99M2 infection was observed after 10 days of inoculation. PXO99^A^ inoculated leaves exhibited the hypersensitivity reaction based on the mode of action of the executor *R* gene in CBB23. Contrastingly, P99M2 inoculated leaves of CBB23 exhibited chlorotic lesions length explaining the situation that *AvrXa23* is mutated in *Xoo*. Transcriptome profiling of CBB23 under infection of PXO99^A^ and P99M2 described that up-regulated genes in PXO99^A^ inoculated samples were down-regulating in P99M2 inoculated leaves of CBB23 at different time periods.

It is established that an oxidative response is an early and complex reaction induced by different biotic stresses and plays diverse roles in plant-pathogen interaction. Peroxidases are important to counter the pathogens in plants by lignification^17,18^, cross-linking of cell wall proteins^19^, programmed cell death and wounds healing etc^20^. In our experiment, six peroxidases related DEGs (Os02g0240300, Os04g0602100, Os07g0677500, Os08g0113000, Os10g0109300, and Os10g0109600) were identified that may have contributed to *Xa23-*mediated resistance against *Xoo*. However, peroxidases activity induce thickening of the secondary wall, hindering the *Xoo* to enter into living cells^21^. *OsPrx114* is exhibited to enhance resistance to necrotrophic foliar pathogens in transgenic carrot^22^. Comparative transcriptome study revealed that peroxidase responsive genes, *PR9*, *PR12*, and *PR14*, were up-regulated in resistance rice cultivar against *M*. *oryzae* infection, illustrating that these peroxidase responsive genes might have significant role in resistance^23^; likewise, the overexpression of *OsAPX8* was claimed to positively regulate resistance against bacterial blight in rice^24^. Peroxidases are believed to be involved in the oxidation of toxic reductants, removal of H_2_O_2_ and defense against the pathogen; also, peroxidases are involved in the crosslinking of the cell wall components, such as pectin and tyrosin, making the cell wall too hard and dense to limit the pathogen entrance into the plant cell^25,26^; Peroxidases involvement in the metabolism of ROS, and reactive nitrogen species activate the hypersensitivity reaction, a type of programmed cell death at the infection site to obviate the pathogen development^27^.

TFs are proteins that act together with other transcriptional regulators, including chromatin remodeling and obstruct RNA polymerase to DNA template; the complexity of the regulation can be reckoned that plant genome assigns approximately 7% of their coding sequence to the TFs^28^. Rice plant induces different TFs, i.e., ERF, WRKY, MYB, NAC, MAD etc., to counter the hazardous biotic stress stimuli. Besides, TFs induce the expression of target genes by binding to the specific promoter site to attain the cellular homeostasis. WRKY TFs are important regulators in rice against biotic stress. In our study, OsWRKY28, OsWRKY45, OsWRKY47, OsWRKY62, OsWRKY69, OsWRKY71, OsWRKY74, and OsWRKY76 were up-regulated at different time periods in PXO99^A^ vs P99M2. Moreover, OsWRKY28 (Os06g0649000) was exhibited to activate the pathogenesis-related gene, *OsPR-10*, contributing to resistance against *Xoo*^29^. OsWRKY47 (Os07g0680400) was found to confer resistance against *M*. *grisea*, BR32 and BR29, via reprogramming, involved in metabolic and signaling pathways^30^. OsWRKY45 (Os05g0322900) was revealed to be a positive regulator of rice plant resistance which responses to *Xoo* and *M*. *oryzae* by modulating the SA and JA levels^31,32^. Unlike, *Xa23-*mediated resistance against *Xoo*, OsWRKY62 (Os09g0417800) was negatively regulated in *Xa21* mediated resistance against *Xoo*^33^. OsWRKY69 (Os08g0386200) was induced after *M*. *grisea* infection but suppressed upon osmotic stress in leaves; whereas, OsWRKY74 (Os09g0334500) was up-regulated after infection by three *M*. *grisea* strains (BR29, BR32, and FR13) and repressed in leaves and roots upon osmotic stress^34^. Overexpression of OsWRKY76 (Os09g0417600) led to the suppression of *PR* genes, involved in phytoalexin synthesis that negatively regulates the blast disease resistance in rice^35^; on the contrary, OsWRKY76 induced the *OsPR-10*, conferring resistance against *Xoo*^29^. Moreover, OsWRKY76 exhibited the positive expression pattern against PXO99^A^ in resistance genotype comparative to the susceptible genotype, JG30, in the previous study^36^.

The expression of MYB TFs was observed after PXO99^A^ and P99M2 infection in CBB23 at different time intervals. Os01g0975300, Os04g0517100, Os05g0114700, Os05g0429900, and Os12g0586300 were observed to be up-regulated in PXO99^A^ relative to P99M2. OsMYB4 (Os04g0517100) was identified to mediate the sheath blight resistance in rice by binding to the promoter region of the oxidoreductase and peroxidase responsive genes^37^. It is confirmed that coordinative activity of MYB48 and MYB59 regulates the JA mediated resistance to necrotrophic pathogens^38^; however, R2-R3 MYB TFs were suggested to enhance resistance to blast infection with less lesion number in inoculated leaves compared to the control plants of rice^39^.

NAC TFs are suggested to have an important role in plant growth, development, abiotic and biotic stress responses^40^. Given the expression of different *OsNAC* genes, it was found that OsNAC9 (Os03g0815100), and OsNAC131 (Os12g0123700) were up-regulated in PXO99^A^ vs P99M2 at different time periods. According to previous studies, OsNAC4 seemed to be a positive regulator of hypersensitive reprogrammed cell death, decreasing the infection caused by different bacterial strains in rice^41^. OsNAC9 appeared to up-regulate the genes involved in lignin biosynthesis, and wall-associated kinases, cell elongation, morphogenesis, and modify the root architecture in rice under drought stress^42^. OsNAC131 was found to be localized in the nucleus and has an important role in rice disease resistance, *M*. *grisea*, and OsNAC131 responses through the regulation of different defense and signaling related genes^43^.

AP2/ERF TFs found only in plants, are involved in different disease resistance pathways. Mainly, ERF responsive genes are induced by pathogen infection, wounds, osmotic stress, hypoxia and drought stress etc^44^. AP2/ERF TFs activate the defense-related genes, *PR* genes, chitinase, and β-1, 3-glucanase^45^. Moreover, in tobacco, OPBP1 enhanced the resistance to the pathogen when expressed ectopically in transgenic rice^46^; OsERF922 negatively regulates the resistance to *M*. *oryzae*^47^. In *Arabidopsis thaliana*, RAP2.2 was revealed to enhance resistance to *Botrytis cinerea*^48^. It is predicted that up-regulated AP2/ERF TFs may be involved in resistance pathways underlying *Xa23*.

The clusters of DEGs related to different KEGG pathways, i.e., “biosynthesis of plant hormone”, “ribosomes”, and “biosynthesis of phenylpropanoid biosynthesis” may be involved in the resistance mechanism against PXO99^A^. Biosynthesis of phenylpropanoid seemed to be important for lignification process, a vital process to protect the plants in abiotic and biotic stresses^14,49^. The phenylpropanoid biosynthesis was observed to be important in rice against *M*. *oryzae* and *Xoo*^23,36^. Previously literature illustrated that ribosome is an enriched pathway in rice and banana against *M*. *oryzae* and *Fusarium oxysporum*, respectively^23,50^.

The plant hormones, i.e., JA, SA, and ET have a crucial role in plant development and immunity. It was mentioned that treatment of JA up-regulates the *PR* genes in rice^51^; furthermore, A key JA enzyme, OsAOS2, which encodes allene oxide synthase exhibited the resistance to *M*. *oryzae*^52^, and increased accumulation of JA was observed in rice, causing resistance to *Xoo*^53^. In our study, the identified SA responsive genes were down-regulated in PXO99^A^ vs P99M2, which exhibited that identified SA responsive DEGs might be down-regulated in disease resistance pathway. Previous literature revealed that unlike mock samples, SA was down-regulated in *Xa7*-mediated resistance to bacterial blight at high temperature^54^. The down-regulation of SA responsive genes might be owing to the antagonistic behavior of the JA and SA in defense signaling pathway^55^. The overexpression of ET responsive gene, *OsACS2*, exhibited the broad-spectrum resistance to various pathogens, e.g., *M*. *oryzae* and *Rhizoctonia solani*^56^. Additionally, another ET responsive gene, *OsEIL2* was suggested to bind to the promoter of *OsrbohA*/*OsrbohB* and *OsOPR4*, having a role in JA biosynthesis and phytoalexins accumulation to counter the *M*. *oryzae* infection^57^.

The cell wall is a complex and protective layer around every plant cell, functioning as a passive defensive barrier. Either by impairing or overexpression of cell wall-related genes exhibited to have an impact on abiotic and biotic stresses^58^. *Xa4* is a resistance gene, conferring resistance to *Xoo* by encoding wall-associated kinase that promotes the cellulose synthesis to maintain the cell wall integrity^59^. Likewise, hemicelluloses are plant cell wall polysaccharides, having β-1,4-linked backbones with an equatorial configuration. Diverse polysaccharides in Arabidopsis, such as xylans and xyloglucans illustrated resistance to necrotrophic fungus *Plectosphaerella cucumerina*^60^. Based on the previously published literature, up-regulated cell wall responsive genes are predicted to be important in *Xa23*-mediated resistance to maintain the cell integrity and restrict the entry of the pathogens into the plant cell.

Hence, this RNA-Seq based transcriptomic analysis led to the identification of genes that were differentially regulated under the PXO99^A^ and P99M2 inoculations at different time points. This comprehensive overview of the DEGs in rice genotype, CBB23, constitutes a valuable resource for researchers aiming to explore the key players in *Xa23*-mediated resistance against *Xoo*.

## Materials and Methods

### Plant materials and Growth Conditions

Initially, seeds of CBB23 were surface soaked using 0.1~0.2% of the potassium permanganate (KMnO_4_) solution for 24 hours and washed with sterilized water. Subsequently, seeds were germinated on a wet filter paper at 37 °C. After emergence, seedlings were transplanted in pots in the greenhouse (25/30 °C under 14 h light/10 h dark cycle with 80% humidity) of Chinese Academy of Agricultural Sciences (CAAS), Beijing, P.R. China, and grown for 2 months.

### Bacterial inoculation and leaf sampling

Two different *Xoo* strains, PXO99^A^ and P99M2, were used for inoculation. P99M9 is a mutant strain of PXO99^A^, without *avrXa23*. Both strains, PXO99^A^ and P99M2, were subcultured on TSA media (tryptophan, 10 g/L; sucrose, 10 g/L; glutamic acid, 1 g/L, and agar, 5 g/200 ml) for 48 hours. The inoculum was prepared by suspending the bacterial strains in sterilized water and concentration was measured by determining the OD_600_ (Optical density at 600 nm) between 0.9 and 1.0. For the observation of disease symptoms, CBB23 leaves were inoculated with PXO99^A^ and P99M2 using scissors dipped method in bacterial suspension to clip the leaves 2–3 cm down from the tip of the leaf blade.

For RNA-Seq, sixty days old leaves (vegetative phase) were selected for PXO99^A^ and P99M2 inoculations through a needleless syringe. The inoculated leaves were harvested with three biological replicates at 12, 24, 36 and 48 hpi, respectively. For getting high-quality RNA, harvested leaves were immediately frozen in liquid nitrogen and stored at −80 °C until RNA extraction.

### RNA extraction and construction of cDNA library for Illumina sequencing

For Illumina sequencing, total RNA from the inoculated (12, 24, 36 and 48 hpi) and mock (C0) leaves of CBB23 genotype was isolated by TRIzol reagent kit (TIANGEN, Beijing, China) according to the manufacturer’s protocol. Later, the samples were purified by RNase free DNase I (TakaRa, Kyoto, Japan) to remove the genomic DNA traces. Total RNA concentration in different samples was calculated using NanoDrop microvolume spectrophotometer (Thermo Scientific NanoDrop Products, Waltham, MA, USA). Then, Illumina HiSeq2500 platform was used for sequencing; library preparation and Illumina sequencing were done by Novogene Bioinformatics Technology Co., Ltd., Beijing, China.

### Data analysis of Illumina sequencing

After Illumina2500 sequencing, quality of raw reads was accessed by fastQC v0.11.2^61^. The low-quality reads and adapters were trimmed by a cutadapt tool^62^. Sometimes, low-quality reads are produced by a sequencing machine that can affect the subsequent bioinformatics analysis. Having trimmed the reads, fastQC was again applied to the reads data to check the reads quality. Each paired-end library has the insert size between 200–300 bp. Thereafter, clean reads were mapped to the available Japonica rice Nipponbare genome using TopHat v2.0.12, by applying Bowtie2 v2.0.0^63,64^. After that, Cufflinks was applied to measure the transcript abundance and expression of each transcript in FPKM (fragments per kilobase pair of exon model per million fragments mapped)^65^. Gene expression differences in the different samples were detected using Cuffdiff. To investigate the genes involved in the resistance and susceptibility of rice, four sample pairs, PXO99^A^ vs. P99M2 (12, 24, 36 and 48 hpi) were used to investigate the DEGs. Actually, Cuffdiff tool tells us the up and down-regulating genes by comparing the expression level of transcripts between two or more conditions. The subsequent list of DEGs was filtered with Log2FC ≥ 1 (up-regulated genes) or ≤*−*1 (down-regulated genes) to determine the significant differences in gene expression.

### Functional classification of DEGs and co-expression analysis

GO functional enrichment analysis was done to identify which DEGs are significantly enriched in GO terms. The GO analysis was carried out by AgriGO software with FDR ≤ 0.05 to get the GO annotations based on biological process (BP), molecular function (MF) and cellular component (CC)^66^. For pathway analysis, we mapped all the DEGs in terms of KEGG and retrieved the significantly enriched pathway with *p*-value ≤ 0.05^67^. Furthermore, MapMan package from the Max Planck Institute of Molecular Plant Physiology, Germany was employed to get the graphical representation of the biotic stress response by DEGs^68^. The co-expression gene networks were developed by RiceFREND^69^; Log2FC values were used to identify the clusters of co-expressed genes, and the co-expression networks were visualized in Cytoscape.

### Validation of RNA-Seq data

To validate the RNA-Seq results, the expression of up and down-regulated genes was confirmed by qRT-PCR. The sequences of randomly selected 10 DEGs were retrieved from Phytozome v12.1^70^. The primers were designed according to the transcript sequence of the genes using AmplifX 1.5.4 software, and the primers used in the qRT-PCR are listed in Supplementary Table 10. Ubiquitin was used as an internal control in qRT-PCR. The reaction was carried out in a 96-wells plate on an ABI prism 7500 Real-Time PCR system (Applied Biosystem, Foster City, CA, USA) using SYBR Green Master ROX (TaKaRa). The thermal cycler conditions were 95 °C for 30 s, followed by 40 cycles of 95 °C for 10 s, 60 °C for 34 s and 72 °C for 15 s. The relative expression level of the selected DEGs was calculated with the 2^−*ΔΔCT*^ method^71^. The qRT-PCR was performed using three biological replicates with three technical replicates.

## Supplementary information

Supplementary table 1-10

Supplementary files

## Data Availability

All the original RNA-Seq data has been submitted to the NCBI Sequence Read Archive under the accession number of SRP154928.
