# Supplementary material for: Transcriptome analysis of a rice cultivar reveals the differentially expressed genes in response to wild and mutant strains of Xanthomonas oryzae pv. oryzae
Source: Sci Rep. 2019 Mar 6;9:3757. doi: 10.1038/s41598-019-39928-2 (PMC6403221; doi:10.1038/s41598-019-39928-2)
Supplement: Supplementary file 2 — Supplementary files [file 41598_2019_39928_MOESM2_ESM.pdf]

Transcriptome analysis of a rice cultivar reveals the differentially expressed genes in response to wild and mutant strains of *Xanthomonas oryzae* pv. *oryzae*

Chunlian Wang[1,+], Rezwan Tariq[1, +], Zhiyuan Ji[1, +], Zheng Wei[1], Kaili Zheng[1], Rukmini Mishra[1] and Kaijun Zhao[1,*]

[1]National Key Facility for Crop Gene Resources and Genetic Improvement (NFCRI), Institute of Crop Science, Chinese Academy of Agriculture Sciences (CAAS), Beijing 100081, China

***** Correspondence: zhaokaijun@caas.cn; Tel.: +86-10-8210-5852; Fax: 86-10-8210-8751

[+] These authors contributed equally to this work.

<u>Supplementary files:</u>

Supplementary Table 1. Description of RNA-Seq paired end data produced by Illumina sequencing.

Supplementary Table 2. Detailed list of 1,235 DEGs at different time points in CBB23 genotype after infection of $PXO99^A$ and P99M2 strains. The significant DEGs were retrieved by Log2FC ≥1 (up-regulated genes) or ≤ -1 (down-regulated genes) as a threshold level. The gene ID, FPKM based expression pattern, and chromosomal position are mentioned in the table.

Supplementary Table 3. Description of differentially expressed TFs in CBB23 after infection of $PXO99^A$ and P99M2 strains at different time points.

Supplementary Table 4. Significantly enriched peroxidase responsive genes in CBB23 after $PXO99^A$ and P99M2 infection.

Supplementary Table 5. Summary of GO enrichment in CBB23 after $PXO99^A$ and P99M2 infection.

Supplementary Table 6. Significant GO terms (FDR ≤ 0.05) of DEGs after infection of $PXO99^A$ and P99M2 strains in CBB23 at different time periods.

Supplementary Table 7. Significant KEGG pathway (*p*-value ≤ 0.05) in CBB23 after $PXO99^A$ and P99M2 infection at different time points.

Supplementary Table 8. MapMan analysis of DEGs involved in host-pathogen interaction pathway in CBB23 genotype. The gene ID, bin code, gene description, and average Log2FC values are presented in the below-mentioned table.

Supplementary Table 9. Co-expression network analysis of nominated differentially expressed TFs with other rice genes.

Supplementary Table 10. List of qRT-PCR primers used for RNA-Seq data validation.
